# Inhibition of Notch1 signaling overcomes resistance to the death ligand Trail by specificity protein 1-dependent upregulation of death receptor 5

**DOI:** 10.1038/cddis.2015.261

**Published:** 2015-10-15

**Authors:** A Fassl, K E Tagscherer, J Richter, J De-Castro Arce, C Savini, F Rösl, W Roth

**Affiliations:** 1 Molecular Tumor Pathology, German Cancer Research Center, Im Neuenheimer Feld 280, 69120 Heidelberg, Germany, and Institute of Pathology, Im Neuenheimer Feld 224, 69120 Heidelberg, Germany; 2 Division of Viral Transformation Mechanisms, German Cancer Research Center, Im Neuenheimer Feld 280, 69120 Heidelberg, Germany

## Abstract

The Notch1 signaling pathway contributes to tumorigenesis by influencing differentiation, proliferation and apoptosis. Here, we demonstrate that inhibition of the Notch1 signaling pathway sensitizes glioblastoma cell lines and glioblastoma initiating cells to apoptosis induced by the death ligand TRAIL. This sensitization occurs through transcriptional upregulation of the death receptor 5 (DR5, TRAIL-R2). The increase in *DR5* expression is abrogated by concomitant repression of the transcription factor Sp1, which directly binds to the *DR5* promoter in the absence of Notch1 as revealed by chromatin immunoprecipitation. Consistent with these findings, Notch1 inhibition resulted in increased *DR5* promoter activity, which was impaired by mutation of one out of two Sp1-binding sites within the proximal *DR5* promoter. Moreover, we demonstrate that JNK signaling contributes to the regulation of DR5 expression by Notch1. Taken together, our results identify Notch1 as key driver for TRAIL resistance and suggest Notch1 as a promising target for anti-glioblastoma therapy.

Notch signaling plays a major role in tumorigenesis by influencing differentiation, proliferation and apoptosis. The components of the pathway comprise four Notch receptors (Notch1–4) and their corresponding membrane bound ligands Delta-like (Dll1, Dll3 and Dll4) and Jagged (Jag1 and Jag2). Ligand binding induces a conformational change in the receptor enabling cleavage by the metalloproteinase ADAM (a disintegrin and metalloprotease). This in turn causes exposure of a second cleavage site and subsequent proteolysis by the *γ*-secretase complex which results in the release of Notch intracellular domain (NICD). NICD translocates to the nucleus and forms a transcriptional complex with C-promoter binding factor 1 (CBF1), Mastermind (MAML) and transcriptional co-activators thereby converting CBF1 from a transcriptional repressor into an activator.^1, 2^ The most prominent NICD target genes belong to the hairy and enhancer of split-1 (HES)^3^- and Hey-family^4^ of transcriptional repressors. Additionally, several other targets including CyclinD1,^5^ c-myc,^6^ p21,^7^ and survivin^8^ were identified, indicating that Notch signaling is involved in tumorigenesis and cell death resistance.

The death ligand tumor necrosis factor-related apoptosis inducing ligand (TRAIL) is a member of the tumor necrosis factor superfamily, which initiates extrinsic cell death via binding to the death receptors DR4 (TRAIL-R1) and DR5 (TRAIL-R2). Upon ligand binding, the death inducing signaling complex (DISC) assembles resulting in the cleavage of pro-Caspase 8 and the initiation of apoptosis.^9^ TRAIL has been shown to selectively kill malignant rather than normal cells with the underlying reasons still being elusive.^10^ Initial clinical trials revealed that treatment with both human recombinant TRAIL and TRAIL-agonistic human antibodies is generally well tolerated and non-toxic.^11^ However, TRAIL resistance of tumor cells has frequently been observed^12^ resulting in an increased interest in the restoration of TRAIL sensitivity by combination therapies.

We previously reported that Notch1 signaling promotes survival of glioblastoma cells via epidermal growth factor receptor (EGFR)-mediated induction of anti-apoptotic Mcl-1.^13^ This EGFR-dependent regulation of the anti-apoptotic Mcl-1 protein results in a decreased susceptibility to activators of both the extrinsic and intrinsic apoptotic pathway which can be overcome by Notch1 inhibition. Within this study, we specifically concentrated on mechanisms of Notch1-dependent regulation of death receptor-mediated apoptosis. We demonstrate that inhibition of Notch1 sensitizes glioblastoma cells to TRAIL-induced cell death by transcriptional upregulation of DR5 expression. Notch1-dependent alteration in *DR5* gene transcription is mediated by the specificity protein 1 (Sp1) transcription factor and involves Jun N-terminal kinase (JNK) signaling. This novel Notch1-Sp1-DR5 signaling pathway could be exploited in future therapeutic approaches for glioblastoma.

## Results

### Notch1 inhibition sensitizes glioblastoma cells for TRAIL-induced apoptosis

**Figure 1:**
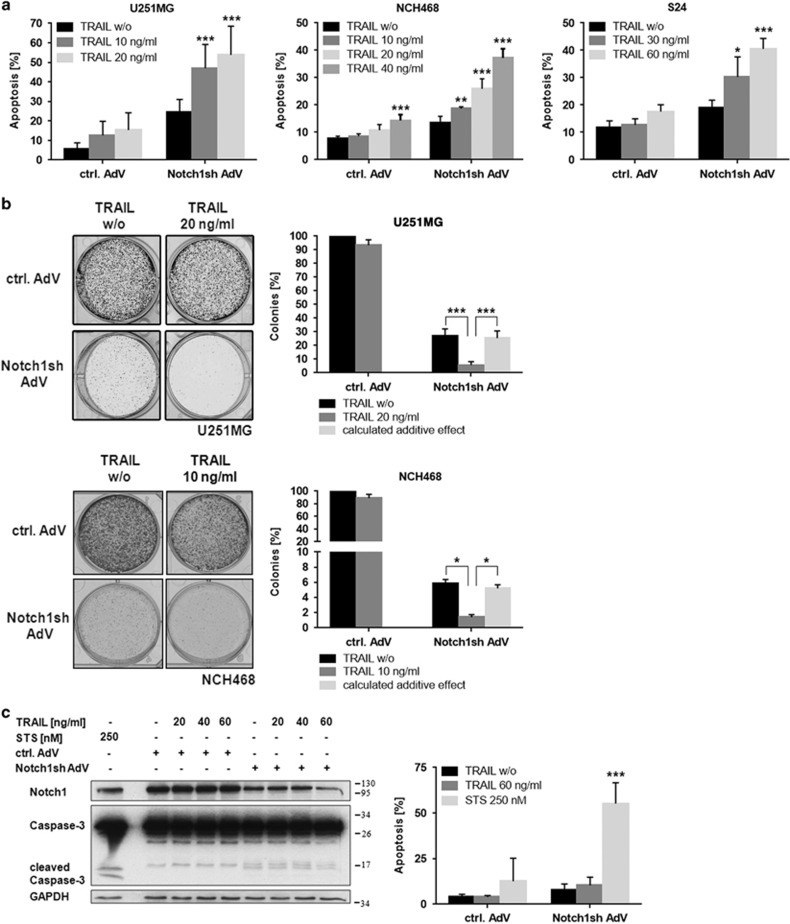
Notch1 inhibition sensitizes glioblastoma cells for TRAIL treatment. (**a**) Glioblastoma cells (U251MG-long-term cell line; NCH468-primary culture; S24-glioblastoma initiating cells) transduced with control-AdV or Notch1sh-AdV (72 h) were treated with TRAIL for 24 h (increased TRAIL concentrations were used for S24 cells because of the high TRAIL resistance of cancer-initiating cells). Apoptotic cell death was measured by flow cytometry (*n*=3; mean ±S.D.). *P*-values were determined by two-way ANOVA. (**b**) Notch1 inhibition together with TRAIL treatment strongly reduces colony formation of glioblastoma cells. Control-AdV and Notch1sh-AdV transduced U251MG and NCH468 cells were seeded in 6-well plates 24 h post transduction (U251MG: 1 × 10^4^ cells; NCH468: 4 × 10^4^ cells). Cells were treated with TRAIL 48 h following seeding and 72 h post transduction, respectively. Colony growth was assessed by crystal violet staining 6 days (U251MG) or 5 days (NCH468) after seeding. Representative staining results are shown (left panels). Colony growth was quantified from three independent assays (right panels). *P*-values were determined by one-way ANOVA test. (**c**) Notch1 inhibition followed by TRAIL treatment does not target astrocytes. Human astrocytes transduced with control-AdV or Notch1sh-AdV (72 h) were treated with varying concentrations of TRAIL or 250 nM STS for 24 h. Total cell lysates were subjected to immunoblot analysis for Notch1 and Caspase-3 (left panel) or apoptotic cell death was measured by flow cytometry (*n*=3; mean±S.D.) (right panel). *P*-values were determined by two-way ANOVA. STS treated astrocytes served as a positive control for Caspase-3 cleavage and apoptosis, respectively

Our previous work suggested an important role of Notch1 in the regulation of apoptosis resistance in glioblastoma cells.^13^ Given the therapeutic potential of TRAIL as a natural anti-tumor agent we sought to elucidate the crosstalk between Notch1 and the extrinsic apoptotic pathway. As a first step, we confirmed the sensitization of glioblastoma cells to TRAIL-induced apoptosis following Notch1 inhibition using long-term cell lines, primary cultures, and glioblastoma initiating cells (Figure 1a). Notch1 knockdown was achieved by an adenovirus delivering a Notch1-specific shRNA. Furthermore, TRAIL treatment in combination with Notch1 downregulation strongly reduced colony formation (Figure 1b). Importantly, human (non-transformed) primary astrocytes were not sensitized to TRAIL by inhibition of the Notch1 pathway (Figure 1c).

### Notch1 signaling regulates expression of the DR5 protein

**Figure 2:**
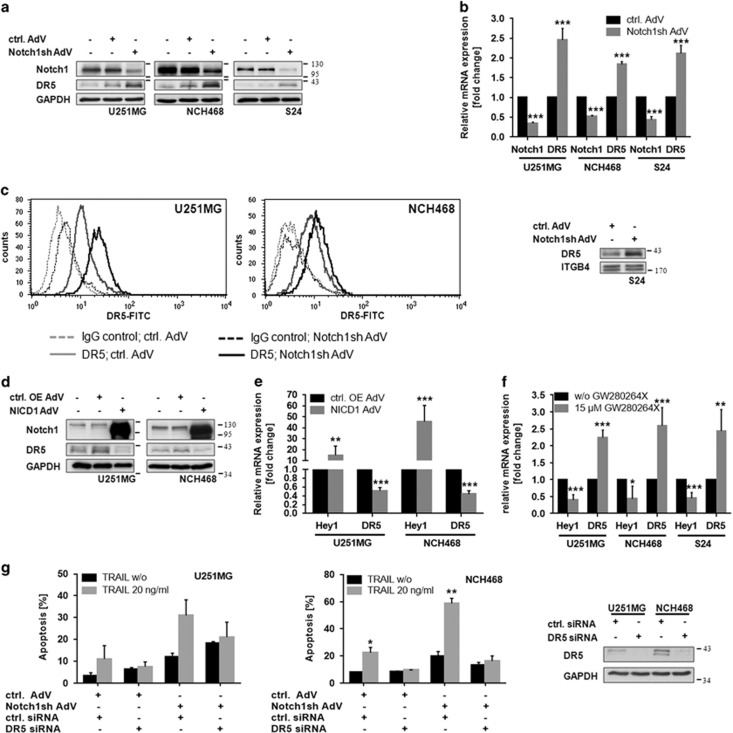
Notch1 signaling regulates the DR5 protein. (**a**) Immunoblot analysis of Notch1 and DR5 in wild type, control-AdV and Notch1sh-AdV transduced U251MG, NCH468 and S24 cells 72 h post transduction. (**b**) Quantitative real-time PCR analysis of Notch1 and DR5 mRNA expression in control-AdV and Notch1sh-AdV transduced glioblastoma cells 72 h post transduction. Expression data were normalized to internal 18 S rRNA expression (*n*=3, mean±S.D.). *P*-values were determined by *t*-test. (**c**) Inhibition of Notch1 signaling increases the amount of DR5 at the cell surface. Amount of DR5 located at the membrane was determined by flow cytometry using a FITC-labeled anti-DR5 antibody and a FITC-labeled IgG as a control (left and middle panel) or by performing membrane fractionation (right panel). For the latter ITGB4 served as a loading control. (**d**) Immunoblot analysis of Notch1 and DR5 in wild type, control OE-AdV (empty), and NICD1-AdV transduced glioblastoma cells 72 h post transduction. (**e**) Quantitative real-time PCR analysis of the Notch1 target gene *Hey1* and DR5 mRNA expression in control OE-AdV (empty) and NICD1-AdV transduced glioblastoma cells 72 h post transduction. Expression data were normalized to internal 18 S rRNA expression (*n*=3, mean±S.D.). *P-*values were determined by *t*-test. (**f**) Quantitative real-time PCR analysis of the Notch1 target gene *Hey1* and DR5 mRNA expression in U251MG, NCH468 and S24 cells following treatment with the ADAM inhibitor GW280264X (15 *μ*M) for 48 h. Expression data were normalized to internal 18 S rRNA expression (*n*=3, mean±S.D.). *P*-values were determined by *t*-test. (**g**) Control-AdV and Notch1sh-AdV transduced U251MG and NCH468 cells (24 h) were transfected with control siRNA and DR5 siRNA, respectively. Cells were treated with TRAIL 48 h post transfection for 24 h. Apoptotic cell death was measured by flow cytometry (*n*=2; mean ±S.D.). *P*-values were determined by *t*-test (left and middle panel). Immunoblot analysis for DR5 48 h post transfection (right panel)

Suppression of Notch1 signaling resulted in a substantial upregulation of the death receptor DR5 as assessed by immunoblot analysis (Figure 2a). Elevation of DR5 protein levels was accompanied by an increase in DR5 mRNA (Figure 2b). Importantly, the increase of DR5 protein after Notch1 knockdown was also detectable at the cell surface as determined by flow cytometry and membrane fractionation, respectively (Figure 2c). Consistently, overexpression of NICD resulted in decreased DR5 protein and mRNA levels (Figures 2d and e). To test whether DR5 upregulation can also be achieved by pharmacological inhibition of Notch activation, we treated cells with the ADAM17 inhibitor GW280264X. ADAM17, together with ADAM10, are the metalloproteinases mediating the initial cleavage of the Notch1 receptor following ligand binding. As expected, treatment with GW280264X resulted in an inhibition of Notch signaling as determined by expression levels of Hey1, a prominent Notch target gene. Importantly, this was paralleled by a substantial increase in DR5 mRNA levels (Figure 2f).

Besides DR5, the death receptor DR4 can also significantly contribute to apoptosis induced by TRAIL treatment in many tumor entities. Although DR4 has been shown to have a minor role in glioblastomas by several studies,^14, 15, 16^ we wanted to exclude any contribution of DR4 to the observed TRAIL sensitization upon Notch1 inhibition. Therefore we tested expression and cell surface density of DR4 in the cell lines used. Although DR4 is almost undetectable in S24 cells (Figure S1A right panel), U251MG and NCH468 cell express DR4 but it is not detectable at the cell surface (Supplementary Figure S1A left panel, S1B). Moreover, Notch1 inhibition has no effect on total protein levels of DR4 in U251MG and NCH468 cells. In line with this, knockdown of DR5 using a DR5-specific siRNA oligonucleotide completely abrogated the sensitization of cells to TRAIL-mediated apoptosis upon Notch1 inhibition (Figure 2g) confirming DR5 as the main signal transducing TRAIL receptor in glioblastomas.

**Figure 3:**
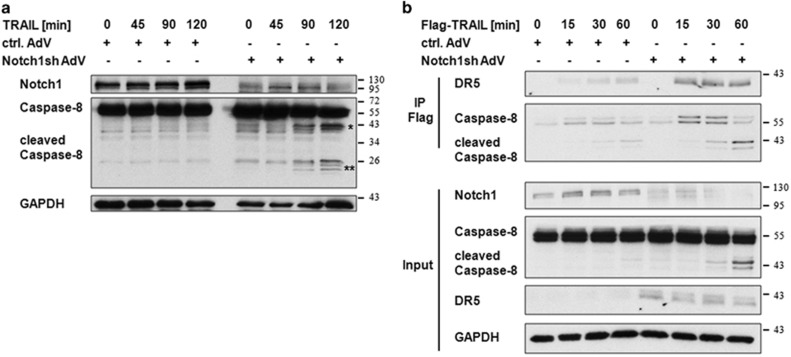
Inhibition of Notch1 signaling causes an increased Caspase-8 cleavage and DISC formation upon TRAIL treatment. (**a**) U251MG cells transduced with control-AdV or Notch1sh-AdV (72 h) were treated with TRAIL (40 ng/ml) for the indicated times. Total cell lysates were subjected to immunoblot analysis for Notch1 and Caspase-8. Asterisk indicate cleaved Caspase-8 fragments (*=p41/p43; **=p24/p26). (**b**) Control-AdV and Notch1sh-AdV transduced U251MG cells (72 h) were treated with Flag-TRAIL (500 ng/ml) for the indicated time points. Subsequently, Flag was immunoprecipitated and immunoblots were probed with DR5 and Caspase-8 antibodies. Whole cell lysates were used for input controls

To further confirm the relevance of DR5 upregulation after Notch1 knockdown for TRAIL-induced apoptosis in glioblastomas, we analyzed the extent of caspase-8 activation and DISC-formation following TRAIL-treatment. Using immunoblot analysis we observed both, a significantly increased caspase-8 cleavage (Figure 3a) and DISC-formation (Figure 3b) upon TRAIL treatment with concomitant Notch1 inhibition.

### Notch1 regulates DR5 via the transcription factor Sp1

To elucidate the mechanism underlying the increased expression of DR5 following Notch1 inhibition we examined the involvement of transcriptional regulators known to induce *DR5* expression. Several previous studies have identified the transcription factor CCAAT-enhancer-binding protein homologous protein (CHOP) as a potent inductor of *DR5* expression following various stimuli in glioma cells.^17, 18^ However, we did not observe an increased CHOP expression upon Notch1 inhibition (data not shown).

**Figure 4:**
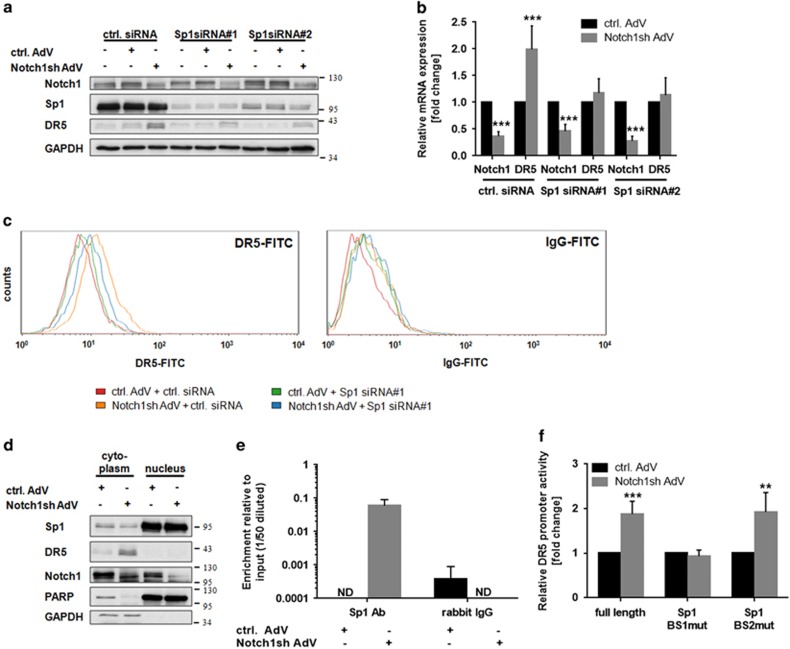
Notch1 regulates DR5 via the transcription factor Sp1. (**a** and **b**) U251MG cells transduced with control-AdV or Notch1sh-AdV were transfected with Sp1 siRNA oligonucleotides 24 h post transduction. (**a**) Immunoblot analysis for Notch1, Sp1, and DR5 72 h post transduction. (**b**) Quantitative real-time PCR analysis of Notch1 and DR5 mRNA expression 72 h post transduction. Expression data were normalized to internal 18 S rRNA expression (*n*=3, mean±S.D.). *P-*values were determined by *t*-test. (**c**) U251MG cells transduced with control-AdV or Notch1sh-AdV were transfected with Sp1 siRNA oligonucleotides 24 h post transduction. Amount of DR5 located at the cell membrane was determined by flow cytometry 48 h post transfection using a FITC-labeled anti-DR5 antibody (left panel) and a FITC-labeled IgG as a control (right panel). (**d**) Cytoplasmic and nuclear levels of Sp1 remain stable following Notch1 knockdown. Immunoblot analysis for Notch1 and Sp1 in cytoplasmic and nuclear lysate fractions from wild type, control-AdV and Notch1sh-AdV-transduced U251MG cells 72 h post transduction. (**e**) Binding of Sp1 to the *DR5* promoter is strongly enhanced following Notch1 downregulation. ChIP analysis of Sp1-binding to the *DR5* promoter in U251MG cells transduced with control-AdV or Notch1sh-AdV (72 h) (*n*=2; mean±S.D.). Rabbit IgG was used as a control for unspecific binding. (**f**) Relative *DR5* promoter activity measured in U251MG cells transduced with control-AdV or Notch1sh-AdV using luciferase reporter constructs containing either the full-length *DR5* promoter (−198 bp) or the *DR5* promoter with mutated Sp1-binding site 1 (Sp1 BS1mut) and mutated Sp1-binding site 2 (Sp1 BS2mut), respectively (*n*=3; mean±S.D.). *P*-values were determined by *t*-test. ND, not detected

Another activator of *DR5* gene expression is the transcription factor Sp1.^19, 20^ However, Sp1-mediated induction of *DR5* expression has not yet been reported in gliomas. To examine a putative role of Sp1 in Notch1-dependent regulation of DR5, we downregulated Sp1 using Sp1-specific siRNA oligonucleotides. Remarkably, concomitant inhibition of Notch1 signaling failed to increase DR5 expression, both on the protein and mRNA level (Figures 4a and b). Moreover, a significantly decreased density of DR5 was observed at the cell surface in cells transfected with Sp1 siRNA on top of Notch1 inhibition (Figure 4c). Of note, the expression level and subcellular localization of Sp1 remained unchanged following Notch1 knockdown (Figure 4d).

To further determine whether Notch1 signaling modulates binding of Sp1 to the *DR5* promoter we performed chromatin immunoprecipitation experiments. Indeed, Sp1 significantly accumulated at the *DR5* promoter upon Notch1 inhibition (Figure 4e) thereby accounting for the increased *DR5* expression. Yoshida *et al.*^21^ described two putative Sp1-binding sites within the proximal *DR5* promoter and both sites have been included in the chromatin immunoprecipitation (ChIP) amplicon used for determination of Sp1-binding to the *DR5* promoter within our study. To further analyze the functional relevance of these sites in the Notch1-mediated regulation of the *DR5* promoter activity we performed reporter gene assays. Although the full-length promoter showed a strong increase in its activity upon Notch1 inhibition, mutation of the Sp1-binding site 1 (the one further away from the start site) completely abrogated *DR5* promoter activation following Notch1 knockdown (Figure 4f). However, induction of the *DR5* promoter remained unchanged when Sp1-binding site 2 (most proximal) was mutated (Figure 4f). Therefore, binding of Sp1 to the Sp1-binding site 1 is necessary and sufficient for Notch1-mediated regulation of *DR5* promoter activity in glioma cells.

### JNK is involved in Notch1-mediated DR5 regulation

**Figure 5:**
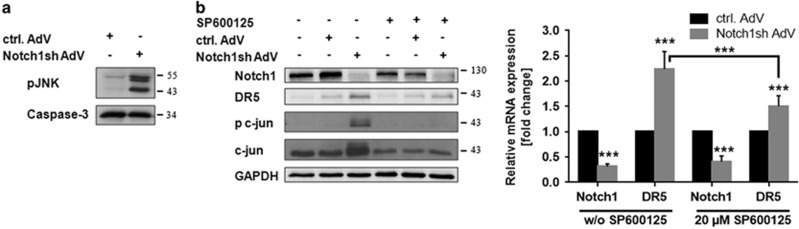
JNK is involved in Notch1-mediated DR5 regulation. (**a**) JNK is activated following Notch1 knockdown. Immunoblot analysis of Notch1 and phospho-JNK in cytoplasmic lysates of control-AdV and Notch1sh-AdV transduced U251MG cells 72 h post transduction. (**b**) U251MG cells transduced with control-AdV or Notch1sh-AdV (48 h) were treated with SP600125 (20 *μ*M) for 24 h. Immunoblot analysis for Notch1, DR5, phospho c-jun and c-jun (left panel). Quantitative real-time PCR analysis of Notch1 and DR5 mRNA expression (right panel). Expression data were normalized to internal 18 S rRNA expression (*n*=3, mean±S.D.). *P-*values were determined by *t*-test (control-AdV *versus* Notch1sh-AdV) or two-way ANOVA (Notch1sh-AdV w/o SP600125 *versus* Notch1sh-AdV 20 *μ*M SP600125)

Increased transcriptional activity of Sp1 towards the *DR5* promoter has formerly been associated with JNK.^19^ Interestingly, we observed that Notch1 inhibition is accompanied by JNK activation in glioma cells (Figure 5a). To examine whether JNK is involved in the upregulation of DR5 following Notch1 knockdown, we blocked JNK activation using the JNK inhibitor SP600125. Concomitant Notch1 and JNK inhibition significantly decreased upregulation of DR5 (Figure 5b) pointing to an involvement of JNK in Notch1-dependent, Sp1-mediated regulation of DR5.

## Discussion

Targeting of death receptors has been proposed as a novel anti-glioblastoma therapeutic approach since the discovery that TRAIL might selectively kill cancer cells. Despite promising preclinical *in vitro* and *in vivo* studies^22, 23, 24^ first clinical trials using TRAIL as a single agent have been disappointing since only modest overall anti-tumor activity was observed in patients with advanced malignancies.^25^ Nevertheless, both recombinant TRAIL and TRAIL receptor agonists have been proven to be non-toxic and well tolerated.^11^ The search for treatment options promoting TRAIL-mediated tumor cell killing is therefore as intense as ever. One major cause for the insufficient response to TRAIL treatment is a strong intrinsic and/or acquired resistance of tumor cells to TRAIL-induced apoptosis. In glioblastomas several resistance mechanisms are known including high expression of TRAIL-decoy receptors,^26^ epigenetic silencing of the TRAIL receptor DR4,^15^ overexpression of the anti-apoptotic protein FLICE-inhibitory protein (FLIP),^27^ and altered expression of the Bcl-2 family members, which negatively interfere with TRAIL-induced apoptosis.^28, 29^

We have previously shown that Notch1 is a central regulator of apoptosis susceptibility in glioblastoma cells.^13^ Here, we analyzed the molecular mechanisms of the strong sensitization of glioblastoma cells to TRAIL-induced apoptosis following inhibition of Notch1 signaling. We identified Notch1 as a mediator of transcriptional repression of *DR5*. In line with our results, inhibition of Notch1 signaling pathway in breast cancer cells has recently been reported to increase the expression of DR4 and DR5 and to enhance the sensitivity to TRAIL-induced apoptosis.^30^ In contrast to these findings, activation of Notch1 signaling increased DR5 expression levels in a p53-dependent manner in hepatocellular carcinoma,^31^ pointing to a cell type dependent regulation.

Transcriptional regulation of *DR5* in glioblastoma has so far been linked to CHOP^17^ and BNIP3 (BCL2 and adenovirus E1B 19 kDa-interacting protein 3)^32^ transcription factors, with the latter functioning as a transcriptional repressor. In other tumor entities, transcription of *DR5* was shown to be activated by nuclear factor *κ*B (NF*κ*B), signal transducer and activator of transcription 1 (Stat1), p53 and Sp1 and repressed by Ying-Yang 1 (YY1).^33^ Although a Sp1-dependent regulation of DR5 was demonstrated in hepatocellular carcinoma,^20, 34^ breast cancer,^35^ colorectal cancer,^36^ and ovary cancer,^37^ a Notch1-mediated regulation of Sp1 and, consequently, the existence of a functional Notch1/Sp1/DR5 axis was so far unknown.

We further evaluated the impact of JNK signaling in Notch1-dependent DR5 regulation. In accordance with our study, modulation of JNK activity by Notch1 has recently been reported.^30^ It has further been proposed, that NICD1 directly binds to and thereby suppresses the scaffold activity of JNK-interacting protein 1 (Jip1) thus preventing the activation of JNK.^38^ JNK was described to phosphorylate the Sp1 transcription factor at Thr278 which increases its stability and prevents its degradation.^39, 40^ Further, JNK-dependent phosphorylation of Sp1 at Thr739 repressed sumoylation of Sp1, thereby blocking the binding of a SUMO-activated E3-ubiquitin ligase and increasing the stability of Sp1.^41^ However, we did not observe altered expression levels of Sp1 upon Notch1 downregulation. Nevertheless, JNK is capable of additionally regulating the activity of Sp1 in an indirect manner without affecting its expression levels. The JNK target c-jun directly binds to Sp1 resulting in an enhanced Sp1 activity.^42, 43^ Further, Sp1 is similarly affected by binding to early growth-response gene product 1 (Egr-1),^44^ which in turn is transcriptionally upregulated by JNK via the activator protein 1 (AP1) transcription factor.^45, 46^ Preliminary results we obtained from ChIP analysis using U251MG cells concomitantly treated with Notch1sh-AdV and the JNK-inhibitor SP600125 point towards this indirect regulation as no difference in Sp1-binding to the *DR5* promoter was observed (data not shown). This would explain why JNK-inhibition is not sufficient to completely abrogate DR5 induction upon Notch1 downregulation. In this scenario, Notch1 knockdown causes relocation of Sp1 to the *DR5* promoter and the concurrent activation of JNK provides a Sp1-binding cofactor increasing its activity. However, further analyses are necessary to conclude on this hypothesis.

Still, the importance of JNK for Sp1-mediated *DR5* transcription is quite controversial. Lin *et al.*^37^ showed that modulation of extracellular-signal-regulated kinase 1/2 (ERK1/2) but not JNK influenced Sp1-mediated *DR5* transcription. In contrast, Higuchi *et al.*^19^ demonstrated that inhibition of JNK abrogated the binding of Sp1 to the *DR5* promoter while modulating the ERK1/2 pathway had only moderate effects within this context. Since both studies were performed in ovarian carcinoma and hepatocellular carcinoma, respectively, a cell type and/or tumor entity specific JNK-dependent regulation of Sp1 is highly probable. Besides regulating DR5 expression in a Sp1-dependent manner, JNK might further be responsible for the transcriptional upregulation of the death receptor by activating the transcription factor Stat1^47, 48^ which has also been linked to the regulation of DR5 expression.^49^

The pathway of Notch1-mediated DR5 regulation described here is of particular importance for glioblastoma cell survival. TRAIL acts as an effector molecule of immune surveillance by various T-cell subpopulations, NK-cells and macrophages and is therefore important for the elimination of cancer cells by the immune system.^50^ Glioblastoma cells are furthermore also targeted by TRAIL expressed by reactive astrocytes.^51, 52^ Since high levels of Notch1 expression are a characteristic feature of glioblastomas,^13^ DR5 protein levels are constantly suppressed. With DR5 being the main signal transducing TRAIL receptor on glioblastoma cells^14, 16^ Notch1 signaling allows escape from immune system-induced apoptosis. This is in contrast to the majority of anti-apoptotic signaling pathways deregulated in glioblastomas including the intrinsic anti-apoptotic action of the Notch1 pathway itself,^13^ which protect cancer cells from apoptosis induced through inherent stresses such as oncogenic stress or hypoxia which are pushing them closer to the edge of dying.^53^

With regard to a clinical application, inhibition of Notch1 signaling combined with recombinant TRAIL or DR5 agonists represents an intriguing treatment strategy. Recombinant TRAIL (dulanermin) and several monoclonal antibodies targeting DR5 are in clinical trials both as single agent and in combination therapies.^11^ However, the effects of a combined inhibition of Notch1 signaling and application of DR5 agonists have so far not been evaluated. Amongst different combination therapies analyzed to date, only conatumumab (an agonistic DR5-antibody) together with the cytostatic agent gemcitabine resulted in a longer progression free survival and overall survival in metastatic pancreatic cancer.^54^ Preclinical analysis revealed a synergistic efficacy of TRAIL and chemotherapy in glioblastoma cell lines and primary glioblastoma cells.^55, 56^ This synergistic effect was based on death receptor upregulation^55, 56^ as well as downregulation of anti-apoptotic FLIP protein.^56^ Interestingly, besides negatively regulating DR5 expression, Notch1 positively regulates Mcl-1,^13, 57^ another important player for TRAIL resistance in glioblastomas,^13, 58^ qualifying Notch1 as an even more promising therapeutic target for glioblastomas.

Currently, the *γ*-secretase inhibitors (GSIs) RO492097 and MK0752 are used in clinical trials for glioblastoma patients (www.clinicaltrials.gov). Within this context, administration of MK0752 as a single agent resulted in a stable disease in glioblastoma patients.^59^ Since GSIs have several side effects including gastrointestinal toxicities and skin disorders, and target other pathways in addition to Notch signaling,^60^ specific Notch1 inhibitors are of particular interest. For instance, antagonistic antibodies against DLL4 (OMP-21M18, REGN421) and against Notch1 (OMP-52M51) are currently evaluated in phase I and II clinical trials with the latter apparently being well tolerated.^60^ Additionally, further agents targeting Notch1 signaling are in preclinical development, amongst those the 'stapled' peptide SAHM1 that aims at interrupting the binding of NICD to MAML thereby inhibiting the Notch transcription factor complex.^60, 61^

Besides having a pivotal role for the success of an experimental therapeutic application of TRAIL or DR5 agonists, inhibition of Notch1 signaling might also be interesting with regard to an efficient TRAIL-mediated immunologic tumor defense. Similarly, anti-tumor vaccines, currently evaluated in clinical trials,^62^ would substantially benefit from an enhanced DR5 expression by the tumor. Of note, Notch1 signaling has furthermore been shown to have an essential role in apoptosis resistance in glioma initiating cells.^13, 57, 63^ Here, we report that inhibition of the Notch1 pathway also increased susceptibility of glioma initiating cells to TRAIL-mediated apoptosis besides sensitizing conventional glioblastoma cell lines. Considering the exceptional high apoptosis resistance of these cells,^64, 65^ this finding is of particular interest and further emphasizes the eligibility of Notch1 as a therapeutic target for glioblastoma patients.

## Materials and Methods

### Materials

Soluble human TRAIL was obtained from Enzo Life Sciences (Loerrach, Germany). Staurosporine (STS) and the JNK inhibitor SP600125 were purchased from Sigma (Deisenhofen, Germany). GW280264X was kindly provided by GlaxoSmithKline (Stevenage, UK). The antibodies used in this study were obtained as follows: anti-Caspase-3 (Imgenex, San Diego, CA, USA); anti-Caspase-8, anti-PARP (BD Pharmingen, Franklin Lakes, NJ, USA); anti-GAPDH, anti-DR4, anti-Notch1, anti-Sp1 (Santa Cruz Biotechnology, San Diego, CA, USA); anti-Integrin *β*4 (ITGB4), anti-pJNK, anti-cJun, anti-phospho-cJun (Cell Signaling, Danvers, MA, USA); and anti-DR5 (Sigma).

### Cell culture

Human glioblastoma long-term cell line U251MG was purchased from the American Type Culture Collection (ATCC, Manassas, VA, USA). NCH468 (primary glioblastoma cells) was kindly provided by Christel Herold-Mende (Department of Neurosurgery, University of Heidelberg, Germany). S24 (glioblastoma initiating cells)^66^ were a kind gift from Wolfgang Wick (DKFZ, Heidelberg, Germany). For the experiments described here, the cells were cultured for no more than eight passages. U251MG and NCH468 cell lines were cultured as described previously.^13^ Glioblastoma-initiating cells (S24) were maintained in HAM'S/F12 (Biochrom, Berlin, Germany) supplemented with 20% BIT (PELO Biotech, Planegg, Germany) 1% penicillin/streptomycin, 1% glutamine (Biochrom), 20 ng/ml EGF (Reliatech, Wolfenbuettel, Germany) and 20 ng/ml bFGF (Reliatech) and cultured at 37 °C in a 5% CO_2_ atmosphere.

Cell lines were regularly tested for the presence of contamination using multiplex cell contamination test provided by the German Cancer Research Center (DKFZ) core facility.^67^

### Transfection

Glioblastoma cells were transiently transfected with siRNA oligonucleotides using Lipofectamine 2000 (Life Technologies, Carlsbad, CA, USA). Sp1 siRNA #1 and #2 were obtained from Thermo Scientific (Waltham, MA, USA, #D-026959-01, #D-026959-02) and used in a concentration of 50 nM. DR5 siRNA was purchased from Life Technologies (#s16756) and used in a concentration of 10 nM. A non-specific siRNA served as a control (Thermo Scientific, #D-001810-01).

### Adenoviral transduction

The recombinant adenoviruses Ad-sh-Notch1 (referred to as Notch1sh AdV) and Ad-sh-scrmbl (referred to as ctrl. AdV) were obtained from SIRION BIOTECH (Martinsried, Germany). The recombinant adenoviruses Ad-mNICD1 (referred to as NICD1 AdV) and Ad-control overexpression (referred to as control OE-AdV) were kind gifts from Mirko HH Schmidt (Institute of Neurology, University of Frankfurt, Germany) and Stefan Herzig (DKFZ) respectively. Directly after seeding, glioblastoma cells were transduced with recombinant adenoviruses using a multiplicity of infection (MOI) of 25-100 (U251MG), 100-300 (NCH468 and S24) or 50–100 (astrocytes).

### Reporter gene assay

DR5 reporter gene constructs (pDR5/-198, pDR5mSp1-1, pDR5mSp1-2)^21^ were kindly provided by Toshiyuki Sakai (Department of Preventive Medicine, Kyoto Prefectural University of Medicine, Kyoto, Japan). Forty-eight hours after adenoviral transduction, cells were cotransfected with Renilla luciferase pRL-SV40 (Promega, Madison, WI, USA) and pDR5/-198, pDR5mSp1-1 or pDR5mSp1-2, respectively. Twenty-four hours post transfection, cell lysates were prepared. Activities of both firefly and Renilla luciferase were determined using the dual luciferase reporter assay system (Promega) according to the manufacturer's instructions.

### Quantitative PCR analysis

Quantitative real-time PCR was performed as described previously.^13^ Following primer pairs were used: 18 S: 5′-CATGGCCGTTCTTAGTTGGT-3′ (forward) 5′-ATGCCAGAGTCTCGTTCGTT-3′ (reverse) Notch1: 5′-GGGCCCTGAATTTCACTGT-3′ (forward) 5′-CGCAGAGGGTTGTATTGGTT-3′ (reverse) DR5: 5′-CAGGTGGACACAATCCCTCT-3′ (forward) 5′-AAGACCCTTGTGCTCGTTGT-3′ (reverse) Hey1: 5′-CGAAATCCCAAACTCCGATA-3′ (forward) 5′-TGGATCACCTGAAAATGCTG-3′ (reverse).

### Flow cytometry

For measurement of cell death, glioblastoma cells were treated as indicated, detached, and stained with Annexin V-FITC (Apoptosis Detection Kit I, BD Pharmingen) according to the manufacturer's instructions.

For measurement of cell surface expression of DR4 and DR5 glioblastoma cells were incubated with cell dissociation buffer (Life Technologies) for 30 min at 37 °C and harvested. Cells were stained with a FITC-labeled anti-DR4 antibody (10 *μ*g/ml, Axxora, Loerrach, Germany), a FITC-labeled anti-DR5 antibody (10 *μ*g/ml; Axxora) or a FITC-labeled IgG1-isotype control antibody (10 *μ*g/ml; Ancell, Bayport, MN, USA) for 60 min at room temperature. Cells were washed, resuspended in PBS containing 0.5% FCS and propidium iodide (1 *μ*g/ml).

Cells were subjected to flow cytometry analysis using a Becton Dickinson FACScalibur cytometer and Cell Quest Software (Heidelberg, Germany).

### Immunoblot analysis

Whole cellular lysate generation and immunoblot analysis was performed as described previously.^13^ For membrane fractionation, cells were processed with a plasma membrane protein extraction kit (BioVision, Zuerich, Switzerland) following the manufacturer's instructions.

### DISC-Immunoprecipitation

Cells were incubated with FLAG-tagged TRAIL (Enzo Life Sciences; 500 ng/ml) for the indicated times, washed with PBS and subsequently lysed in lysis buffer (20 mM Tris-HCl, pH 7.4; 150 nM NaCl; 0.2% Nonidet P40; 10% Glycerol; protease and phosphatase inhibitor cocktail (Life Technologies)). The TRAIL-DISC was immunoprecipitated from precleared lysates overnight with 20 *μ*l anti-FLAG antibody M2-conjugated agarose beads (Sigma). Before immunoblot analysis, beads were recovered by centrifugation and washed four times with lysis buffer.

### Chromatin-Immunoprecipitation

A total of 1 × 10^7^ U251MG cells were transduced with recombinant adenoviruses. Seventy-two hours post transduction the cells were cross-linked with 1% formaldehyde and chromatin fractions were prepared exactly as described previously.^68^ After clearance of non-specifically bound chromatin fragments, supernatants equivalent to 25 *μ*g DNA were incubated with 2 *μ*g of Sp1 antibody or 2 *μ*g of rabbit IgG antibody as control (both provided in the same kit; Millipore, Billerica, MA, USA, #17-601; Lot: JBC1920619). Next day, the immune-selected chromatin was eluted from the protein A/G agarose beads adding 250 *μ*l of elution buffer (1% SDS, 100 mM NaHCO_3_), diluted 1 : 1 in de-crosslinking buffer (320 mM NaCl, 80 mM Tris pH 8, 20 mM EDTA pH 8 and 100 *μ*g/ml proteinase K) and incubated overnight at 65 °C. Real-time PCR analysis of immunoprecipitated chromatin was done using the following DR5 promoter-specific primers: forward 5′-AGGTTAGTTCCGGTCCCTTC-3′ reverse: 5′-CGCGTGCTGATTTATGTGTC-3′.^19^ PCR products were quantified relative to the control using rabbit IgG for unspecific binding.

### Colony formation assay

To assess colony formation cells were seeded in 6-well plates (U251MG: 1 × 10^4^ cells; NCH468: 4 × 10^4^ cells) and incubated for five days (U251MG) and six days (NCH468), respectively before crystal violet staining. Colony count was determined using the Clono Counter^69^ software. Synergy was evaluated as described previously.^70^

### Statistical methods

Significant differences were identified by two-way ANOVA, one-way ANOVA or *t*-test. The test used in a certain analysis is stated in the respective figure legend. Whenever necessary *P*-values were corrected for multiple testing using the Holm–Sidak test. Throughout, *P*-values<0.05 were considered significant and are indicated as follows: *P<0.05, **P<0.01, ***P<0.001.

## Abbreviations

ADAM: a disintegrin and metalloprotease
AP1: activator protein 1
ATCC: American type culture collection
BNIP3: BCL2 and adenovirus E1B 19 kDa-interacting protein 3
CBF1: C-promoter binding factor
ChIP: Chromatin immunoprecipitation
CHOP: CCAAT-enhancer-binding protein homologous protein
DISC: death inducing signaling complex
DLL: Delta-like
DR4: death receptor 4
DR5: death receptor 5
EGFR: epidermal growth factor receptor
Egr-1: early-growth-response gene product 1
ERK1/2: extracellular-signal-regulated kinase 1/2
FLIP: FLICE-inhibitory protein
GSI: *γ*-secretase inhibitor
HES: hairy and enhancer of split 1
ITGB4: integrin *β*4
Jip1: JNK-interacting protein 1
JNK: Jun N-terminal Kinase
MAML: mastermind
MOI: multiplicity of infection
NF*κ*B: nuclear factor *κ*B
NICD: Notch intracellular domain
SD: standard deviation
Sp1: specificity protein 1
Stat1: signal transducer and activator of transcription 1
STS: Staurosporine
TRAIL: tumor necrosis factor-related apoptosis inducing ligand
YY1: Ying-Yang 1
