# Supplementary material for: Inhibition of Notch1 signaling overcomes resistance to the death ligand Trail by specificity protein 1-dependent upregulation of death receptor 5
Source: Cell Death Dis. 2015 Oct 15;6(10):e1921–. doi: 10.1038/cddis.2015.261 (PMC4632291; doi:10.1038/cddis.2015.261)
Supplement: Supplementary Information [file cddis2015261x1.pdf]

Fig. S1 - Fassl et al.

A

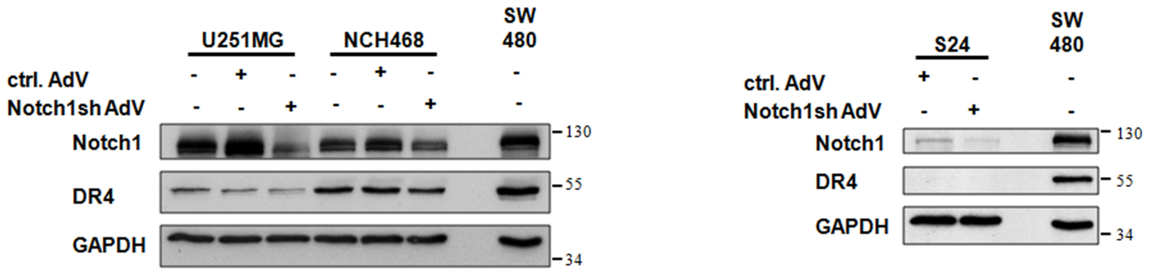

B

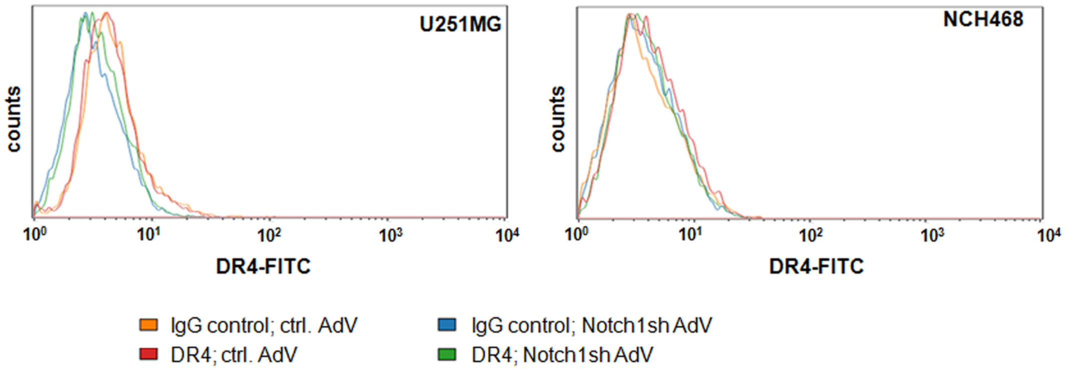

Figure S1: DR4 expression is unchanged following Notch1 inhibition. A. Immunoblot analysis of Notch1 and DR4 in wildtype, control-AdV, and Notch1sh-AdV transduced U251MG and NCH468 cells (left panel) and control-AdV and Notch1sh AdV transduced S24 cells (right panel) 72 h post transduction. SW480 cells serve as positive control. **B.** Amount of DR4 located at the membrane was determined by flow cytometry using a FITC-labeled anti-DR4 antibody and a FITC-labeled IgG as a control.
